# Feasibility and Acceptability of a Prevention-Focused Screener for Perinatal Depression Risk: Mixed Methods Cohort Study

**DOI:** 10.2196/81638

**Published:** 2026-05-05

**Authors:** Tamar Krishnamurti, Samantha Rodriguez, Leah Cope, Lara Lemon, Priya Gopalan, Cara Nikolajski, Hyagriv Simhan, Kelly Williams

**Affiliations:** 1 General Internal Medicine, University of Pittsburgh School of Medicine, 200 Meyran Avenue, Parkvale Building, Suite 300, Pittsburgh, PA, 15213, United States, 1 412-383-5556; 2 Obstetrics, Gynecology, and Reproductive Sciences, University of Pittsburgh School of Medicine, Pittsburgh, PA, United States; 3 UPMC Center for High Value Healthcare, Pittsburgh, PA, United States; 4 Department of Psychiatry, University of Pittsburgh School of Medicine, Pittsburgh, PA, United States

**Keywords:** prediction algorithm, machine learning, preventive care, predictive screening, patient-centered care, depression, pregnancy, postpartum, mental health

## Abstract

**Background:**

More than 20% of perinatal women experience depression, with suicide being a leading cause of maternal death in the United States. Professional societies emphasize the need to identify those at risk of developing perinatal depression to better target preventive care delivery during pregnancy.

**Objective:**

We evaluated receptivity to a machine learning–based predictive screener designed to identify women in the first trimester of pregnancy who were asymptomatic but were at risk for developing moderate to severe depression symptoms later in pregnancy.

**Methods:**

Our participants were adult pregnant women with negative first-trimester depression (Patient Health Questionnaire–9) screens at 1 of 4 obstetric practices. Of the 810 women who were clinically eligible, 787 were successfully contacted via their patient portal. Of these, 289 (36.7%) viewed the screener and 255 (88.2%) completed the 6-question predictive screener. In total, 51 (20%) were identified by the screener as being at risk for developing perinatal depression. Participants were asked a series of follow-up questions regarding the acceptability of the predictive screener and desired preventive resources. Chi-square tests were used to compare demographic characteristics, perceived benefits and concerns, and desired resources between those identified as at risk for depression and those who were not. Differences in acceptability ratings between the two risk groups were determined using nonparametric Mann-Whitney *U* tests.

**Results:**

On a 5-point Likert scale of agreement, participants found the screener questions easy to complete (median score 5, IQR 5-5) and felt comfortable sharing their answers with their obstetric care providers (median 5, IQR 4-5). Key perceived benefits of completing the screener included opportunities to seek preventive care (75/255, 29.4%) and to receive education on depression risk (66/255, 25.9%). Primary concerns about knowing one’s risk of future depression included worrying about developing depression (90/255, 35.3%) and a lack of prevention opportunities (39/255, 15.3%). Desired preventive resources included counseling (197/255, 77.3%), mind-body interventions (166/255, 65.1%) such as exercise, and prenatal classes or support groups (81/255, 31.8%).

**Conclusions:**

Participants found the screener acceptable and felt comfortable receiving it through their patient portal. Specific preventive care options were commonly endorsed, several of which are scalable and evidence based. A minority of participants voiced addressable concerns about knowing their risk of developing depression in the future.

## Introduction

More than 20% of US mothers experience depression during pregnancy and the postpartum period, which is consistent with global rates [1,2]. Untreated depression can contribute to long-term health problems [3-5], including cognitive and emotional challenges for children and unhealthy maternal behaviors, such as alcohol or drug use, insufficient physical activity, and poor diet [6,7]. Furthermore, 1 in 5 women with perinatal depression expresses thoughts of self-harm, with suicide being a leading cause of maternal death [8]. While 40% to 50% of women with perinatal depression have a history of depression, the remainder experience depression onset for the first time during their pregnancy or in the early postpartum period [9,10]. Currently, the United States Preventive Services Task Force recommends early identification of perinatal women at risk of developing depression to facilitate preventive rather than reactive care [11-13]. However, implementing this recommendation requires accurate identification of risk prior to depression onset. This identification is particularly challenging for those without a history of depression, who may not recognize and self-report the onset of early symptoms.

There are documented factors associated with perinatal depression, including pregnancy-related stressors (eg, financial instability, fear of the birth experience, and body image concerns); psychosocial stressors (eg, lack of social support, adverse life events, history of anxiety, and food insecurity); physical health conditions (eg, gestational diabetes and hypertension); and race or ethnicity (eg, Black and Latinx individuals). Nevertheless, little is known about which combination of factors most accurately predicts future risk of developing perinatal depression. Moreover, current perinatal depression screening tools administered during prenatal care are designed to quantify active depression symptoms and do not identify risk factors for future depressive episodes [14,15].

In response to this gap, we developed an interpretable machine learning–based predictive screener that can be administered directly to patients in the first trimester to identify those at risk of developing depression for the first time during pregnancy [16,17]. This 6-question predictive screener was designed to identify individuals who will develop new-onset depression among those who are currently asymptomatic and have no history of depression. The screener was co-designed with clinicians and people with lived experience through an iterative process, including focus groups with providers (eg, obstetricians, family medicine physicians, and behavioral health professionals) and virtual community engagement studios with pregnant and postpartum women, the details of which are described in a prior publication [14]. The predictive screener was shown to have a high degree of accuracy (area under the receiver operating characteristic curve=0.93), sensitivity (0.90), and specificity (0.81) [17]. However, the ultimate usefulness of this risk screening tool will be in its adoption into clinical workflow and its acceptability to the patients completing it. To fully close the loop, successful implementation also requires the provision of preventive care in response to a positive screener score.

A review encompassing 19 systematic reviews and meta-analyses and 152 randomized controlled trials across 26 countries identified therapy-based or physical activity interventions as having a small to moderate effect on the severity of perinatal depression symptoms [18]. Interpersonal and cognitive behavioral therapy are similarly effective for perinatal depression prevention [19-22]. Earlier therapeutic intervention can more effectively address perinatal mental health risk. With developments in predictive screening, there is an opportunity to tailor preventive care to address immediate, pressing risk factors (eg, food insecurity, labor and delivery fears, or lack of social support), while also developing strategies for long-term resilience to manage new and ongoing stressors. However, to date, patient-preferred preventive care options for those at risk of developing perinatal depression have not been documented.

The majority of women receiving prenatal care in our clinical setting access their health care information through a patient portal. As such, deployment of prenatal risk screeners through the patient portal is our standard workflow and is increasingly common in obstetric practices nationally [23-26]. Here, we examined the feasibility of deploying our predictive depression screener through our digital patient portal, as well as patients’ receptivity to completing it. To do this, we deployed the screener to women receiving first-trimester prenatal care in our health care system, followed by a survey on their experience with completing it. We also solicited their preferences for preventive care resources.

## Methods

### Study Design and Setting

Participants were women (aged ≥18 years) receiving prenatal care from 1 of 4 University of Pittsburgh Medical Center obstetric practices. Electronic health records in the participating practices were screened for study eligibility on a weekly basis between October 2024 and January 2025. Eligibility criteria included being in the first trimester of pregnancy and screening negative for active first-trimester depressive symptoms using the Patient Health Questionnaire–9 [15].

Eligible pregnant women were sent an invitation via their patient portal to complete the 6-question predictive screener and subsequent survey questions related to the screener. The invitation contained a unique survey link that expired at the end of the first trimester. Upon accepting the link and completing informed consent, a pregnant woman was considered a study participant. Participants were not provided with a risk score. This allowed for independent validation of the screener in this population through passive observation of depressive symptoms and diagnoses over the course of the pregnancy. However, mental health and other pregnancy resources were provided to all participants, regardless of risk, to reflect best clinical practice.

### Measures

Feasibility of screening was assessed by measuring (1) the proportion of pregnant women eligible from the larger clinical population (*eligibility rate*), (2) the number of eligible individuals who engaged with the screener study invitation (*reach*), and (3) the number of completed surveys among those who accessed the survey (*completion rate*).

Acceptability was assessed with a series of Likert-scale questions (1=strongly disagree to 5=strongly agree), which included participant evaluations of screener question clarity (“The screener questions were clear”), ease of completion (“The screener was easy to complete”), and comfort with and honesty in disclosing responses (“I would be comfortable sharing my answers with my provider” and “I would have no concerns about answering the questions honestly,” respectively).

Participants were asked about the usefulness of the screener as part of routine prenatal care, including the benefits of knowing the results of the screener (“What would be the *benefit(s) to you personally* of knowing you are likely to develop depression at some point during your pregnancy?”), their preferred method of receiving screener results (“If you completed a screener like this as part of your prenatal care, *how* would you prefer to receive your results?”), perceived helpfulness of knowing depression risk (“How *helpful* would it be to know whether you are likely to develop depression at some point during your pregnancy?”), and any concerns they might have if they were to learn that they were likely to develop depression at some point during their pregnancy (“What *concern(s) would you personally have* about knowing you are likely to develop depression at some point during your pregnancy?”). Participants were also asked to endorse from a prespecified list and/or offer open-ended options for desired preventive care resources (“If you learned you were likely to develop depression at some point in your pregnancy, which of the following resources would you be *most likely to use* if available to you at *low or no-cost*?”) and offer any additional thoughts or comments they have about the survey or depression prevention (“We welcome any other thoughts you have about your participation in this survey or preventing depression in pregnancy.”). Deployment feasibility and acceptability of the screener were the primary outcomes of this study. Secondary analyses examined differences in attitudes between high- and low-risk pregnant women.

### Statistical Analysis

Descriptive analyses used means and SDs for continuous variables (eg, maternal age), medians and IQRs for ordinal variables (eg, Likert-scale responses), and distributions for categorical variables. The study sample was stratified by screener score into those with and without risk of developing moderate to severe depression symptoms. Differences in survey responses by risk category were assessed using chi-square tests for categorical and binary responses. The distributions of continuous and Likert-scale responses were assessed for normality using the D’Agostino-Pearson test. Differences between normally distributed responses were evaluated using 2-sample 2-tailed *t* tests, and nonnormally distributed responses were evaluated using Mann-Whitney *U* tests. Effect size was calculated using Cliff *d*, a robust nonparametric method appropriate for ordinal data such as Likert-scale data [27]. An α level of .05 was used for statistical significance. All respondents completed every survey item, resulting in no missing data. Analyses were performed using Python (version 3.12.4).

Rapid inductive thematic coding was applied to open-ended survey responses by 2 coders, each with qualitative coding experience and one with formal training in rapid qualitative analysis. After reading all open-ended survey responses, the coders cocreated a codebook. Responses were then independently coded. Once independent coding was complete, codes were compared, and discrepancies were discussed until consensus (ie, consistent classification) was reached.

### Ethical Considerations

All study procedures were approved by the University of Pittsburgh Institutional Review Board (STUDY24020098) and are reported according to the Strengthening the Reporting of Observational Studies in Epidemiology (STROBE) guidelines for reporting observational studies [28]. We conducted pretests of the survey with pregnant or recently pregnant colleagues, gathering feedback on the design and clarity of the instructions and survey questions. Participants provided digital consent for study participation and were informed that they could withdraw their consent at any time. They received a US $20 gift card for completing the screener and follow-up survey, which took approximately 10 minutes. All participant data were stored in a confidential and secure manner and access was restricted to authorized members of the research team.

## Results

### Survey Participants: Reach and Risk

During the study period, 1925 women received prenatal care at one of the participating obstetric practices. Of these 1925 women, 810 (42.1%) had an active patient portal, were at a gestational age of <14 weeks, and had scored <5 on the Patient Health Questionnaire–9 measure of active depression symptoms (*eligibility rate*). Of the 810 sent an invitation through their patient portal, only 2.8% (n=23) did not access it. In total, 36.7% (289/787) who accessed the invitation opened the study link (*reach*) and, of those, 88.2% (255/289) completed it (*completion rate*).

All participants were female with a mean age of 32 (SD 4.5) years. Most were White (217/255, 85.1%) and non-Hispanic (232/255, 91%), married (196/255, 76.9%), and had commercial insurance (196/255, 76.9%). Full demographic information is available in Table 1. Participants were demographically representative of the larger eligible population.

**Table 1.** Demographic characteristics of participants (n=255).

Demographics		Study sample
Age (years), mean (SD)		32 (4.5)
Gestational age (days), mean (SD)		71 (11.0)
Nulliparous, n (%)		111 (43.5)
Household income (US $), n (%)		
<25,000		23 (9)
25,000-54,999		29 (11.4)
55,000-99,999		37 (14.5)
100,000-149,999		80 (31.4)
>150,000		85 (33.3)
Prefer not to answer or missing		1 (0.4)
Poverty line^a^, n (%)		
Above		193 (75.7)
Below		47 (18.4)
Uncertain^b^		15 (5.9)
Race^c^, n (%)		
American Indian or Alaska Native		1 (0.4)
Asian		10 (3.9)
Black		16 (6.3)
White		217 (85.1)
Multiracial		9 (3.5)
Unknown		0 (0)
Other		5 (2)
Prefer not to answer or missing		5 (2)
Ethnicity, n (%)		
Hispanic or Latinx		11 (4.3)
Non-Hispanic		232 (91)
Unknown		3 (1.2)
Prefer not to answer or missing		9 (3.5)
Marital status, n (%)		
Single or never married		37 (14.5)
Married or domestic partnership		205 (80.4)
Divorced or separated		6 (2.4)
Other		7 (2.7)
Employment status, n (%)		
Employed full-time		172 (67.4)
Employed part-time		27 (10.6)
Stay-at-home parent		29 (11.4)
Seeking employment		7 (2.7)
Not working		14 (5.5)
Other		6 (2.4)
Insurance type, n (%)		
Commercial		196 (76.9)
Medicaid		44 (17.2)
Self-pay or other		15 (5.9)

a These thresholds are calculated using income and household size and are based on 215% of the federal poverty line in 2024, which is a standard threshold for receipt of federal or state assistance programs.

b Uncertain poverty line labels are for participants whose selected income bracket and household size contain the poverty line value for the given household size.

c Race options are not mutually exclusive; therefore, totals exceed 100%.

On the basis of established cutoff scores [17], 80% (204/255) of participants were identified by the predictive screener as having no or minimal depression risk and 20% (51/255) were identified as at risk of developing moderate to severe depression at a later stage of pregnancy.

### Acceptability

The predictive screener demonstrated high acceptability among participants. Most agreed or strongly agreed (236/255, 92.5%) that the screener questions were clear (median 5, IQR 4-5), and 97.3% (248/255) reported that the questions were easy to complete (median 5, IQR 5-5). Most (236/255, 92.5%) agreed or strongly agreed that they would feel comfortable sharing the results of the screener with their obstetric care team providers (median 5, IQR 4-5). Similarly, the majority (247/255, 96.9%) agreed or strongly agreed that they would have no concerns about answering the questions honestly (median 5, IQR 5-5).

Table 2 presents acceptability metrics stratified by depression risk category, with those at risk of developing moderate-to-severe depression symptoms being less likely to endorse strong agreement with comfort and ease of honesty in responding to the screener.

**Table 2.** Median (IQR) responses to acceptability questions rated on a 5-point Likert scale.

Acceptability statements	No or minimal depression risk^a^, median (IQR)	Moderate to severe depression risk^b^, median (IQR)	Mann-Whitney *U* statistic	Effect size (Cohen *d*)	*P* value
The screener questions were clear.	5 (4-5)	5 (4-5)	4413.5	−0.15	.04
The screener was easy to complete.	5 (5-5)	5 (4-5)	4337.5	−0.17	.07
I would be comfortable sharing my answers with my provider.	5 (4-5)	4 (4-5)	3939.0	−0.24	.01
I would have no concerns about answering the questions honestly.	5 (5-5)	5 (4-5)	4218.5	−0.19	.04

a No or minimal depression risk: n=204.

b Moderate to severe depression risk: n=51.

### Perspectives on Receiving Depression Screening Scores

Overall, 80% (204/255) reported that the screener was helpful or extremely helpful (median 4, IQR 4-5). Participants largely voiced a preference for receiving screener results through the patient portal (150/255, 58.8%), compared to receiving results in person (76/255, 29.8%) or by phone (13/255, 5.1%). Perspectives did not differ by depression risk.

### Perceived Benefits and Risks of Depression Risk Screening

When asked about potential benefits of receiving their depression risk screening score, participants most frequently cited seeking appropriate preventive resources and/or treatments (63/204, 30.9%) and the opportunity to receive education from their obstetric health provider (53/204, 26%). One survey participant wrote that the feedback could help them “reduce risk factors associated with postpartum depression and postpartum psychosis.”

Despite generally positive attitudes toward score disclosure, participants also expressed concerns. The most frequently reported concerns included increased anxiety or worry about developing depression (71/204, 34.8%) and worry about the inability to prevent depression (34/204, 16.7%). Several open-ended responses were offered, including concern about parenting ability and emotional connection to children if depression were to occur, premature medication use, and false positives.

Participants at risk of developing moderate to severe depression were significantly more likely than those with no or minimal depression risk to endorse the concern “fear that your health care provider will view you negatively” (5.9% vs 0.5%; *P*=.03). However, at-risk participants were also more likely to cite the ability to identify factors that might be affecting their mood as a benefit (17.6% vs 5.9%; *P*=.01). Table 3 presents the complete breakdown of perceived benefits and risks.

**Table 3.** Breakdown of perceived benefits and risks, stratified by depression risk.

Benefits or concerns		Participants with no depression risk^a^, n (%)	Participants with depression risk^b^, n (%)	*P* value^c^
Benefits				
Allows you to seek appropriate preventive resources (ie, community resources and/or preventive treatments)		63 (30.9)^d^	12 (23.5)	.39
Provides an opportunity to receive education on pregnancy and postpartum depression, symptoms, and/or warning signs from your health care provider		53 (26.0)	13 (25.5)	.99
Provides information to discuss with your support system (eg, partner, friends, and family) about your needs		29 (14.2)	8 (15.7)	.96
Offers insight into why you might be feeling a certain way		26 (12.7)	4 (7.8)	.47
Helps you identify factors that might be affecting your mood		12 (5.9)	9 (17.6)	.*01^e^*
Gives you and your health care provider an opportunity to discuss depression risk		15 (7.4)	4 (7.8)	.99
Another benefit (open-ended)		1 (0.5)	0 (0.0)	.99
Concerns				
Increased anxiety and/or worry about developing depression		71 (34.8)	19 (37.3)	.87
Worry about your ability to prevent depression		34 (16.7)	5 (9.8)	.32
Fear that the results will be reported to child protective services		10 (4.9)	7 (13.7)	.052
Fear that others (eg, partner, friends, and family) will view you negatively		8 (3.9)	5 (9.8)	.18
Inability to access the right preventive resources		10 (4.9)	3 (5.9)	.99
Concern that your health care provider will doubt your parenting abilities		8 (3.9)	1 (2.0)	.80
Fear that your health care provider will view you negatively		1 (0.5)	3 (5.9)	.*03*
Concern that your provider will not know the appropriate education or resources to offer you		1 (0.5)	2 (3.9)	.19
Another concern (open-ended)		6 (2.9)	1 (2.0)	.99

a Participants with no depression risk: n=204.

b Participants with depression risk: n=51.

c *P *values are determined using the chi-square test to compare perceived benefits and concerns between those with and without first-time onset depression in our sample.

d Perceived benefits and concerns are not mutually exclusive; therefore, totals exceed 100% of participants.

e Italicized p-values indicate a statistically significant difference between those with no depression risk and those with depression risk.

### Desired Preventive Care Resources

When asked about desired preventive health resources, participants endorsed multiple options. Preferred resources included counseling (197/255, 77.3%), with 61.8% (126/197) endorsing in-person therapy and 41.6% (82/197) endorsing app-based delivery. Mind- or body-based interventions (166/255, 65.1%) were frequently endorsed, with “yoga, exercise classes, or gym membership” (114/166, 68.7%) being a highly desired intervention, followed by prenatal classes or support groups (81/255, 31.8%).

Participants (n=61) shared several resources not otherwise listed in open-ended responses. From most to least frequently listed, these included mental health resources for their spouse or support person (eg, “Education for family/spouses so they can possibly help the individual battling depression”), self-care opportunities (eg, “massage”), practical support such as cleaning services or meal preparation (eg, “A service similar to task rabbit that is geared towards pregnancy needs. Perhaps people that could help identify inexpensive sources of second hand furniture/items, assembly help, specific errand support, meal prep/planning for once baby arrives, etc.”), parental leave, increased medical touchpoints during pregnancy, additional mental health education (eg, books and fact sheets), medication for depression prevention, structural changes (eg, improved health care access and transportation), access to a live mental health support person (“Mental health support chat forums with a licensed counselor”), a more holistic approach to health care, and additional faith or spiritual support.

**Figure 1.**
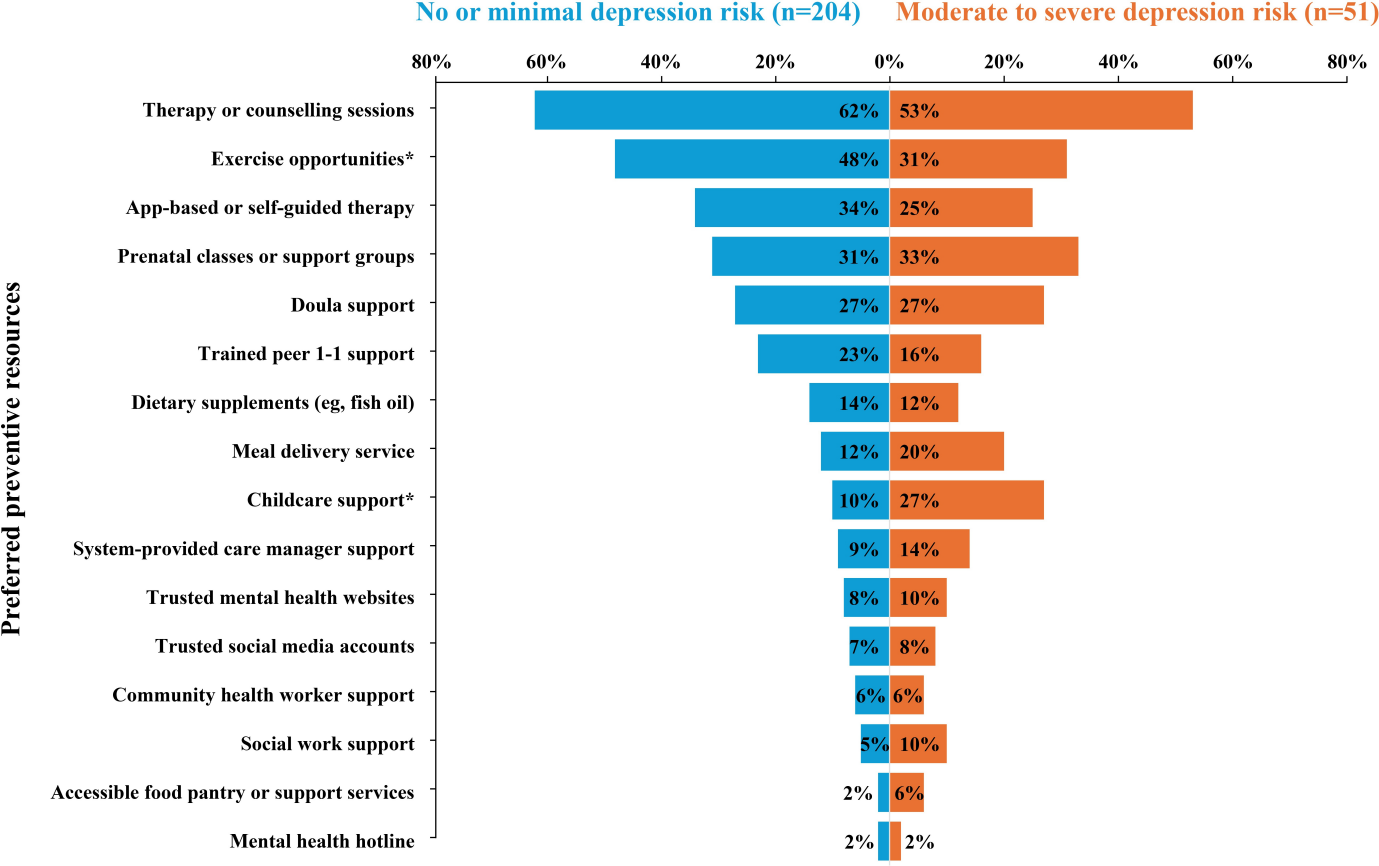
Preferred resources stratified by depression risk. ^*^Substantial differences in resource desirability.

Figure 1 shows preferred resources stratified by depression risk. Notably, less than 10% (20/204) of those with no or minimal depression risk expressed interest in childcare support compared to more than 25% (14/51) of those at risk of developing moderate to severe depression (*χ*^2^_1_=9.52; n=255; *P*=.002). Although highly endorsed by both risk groups, participants with no or minimal depression risk expressed greater interest in exercise opportunities (*χ*^2^_1_=3.94; n=255; *P*=.05).

### Open-Ended Feedback

After completing the survey, 55.3% (141/255) of participants shared optional thoughts about screener participation and about preventing depression in pregnancy. The most frequent open-ended comments expressed appreciation for the study or confirmed that participants would find a screener of this kind helpful. Additional responses ranged from suggestions for the screener or resources (eg, “A big area of concern for working moms is their jobs. I felt that the questions did not address the concern as much.”) to specific stressors (eg, “I think one of the biggest stressors that can contribute greatly to mood in the first trimester is the viability and health/wellness of the baby, i.e., risk of miscarriage or is there any genetic or chromosomal issue?”) and barriers to care (“I feel like cost is one of the biggest barriers to seeking help, just ahead of the stigma that still exists.”). One participant reported feeling that a discussion of depression may exacerbate depressive symptoms. Those at risk of developing moderate to severe depression more frequently expressed the need for help and social support during pregnancy, while those with no or minimal depression risk largely highlighted the need for depression- and anxiety-specific resources.

## Discussion

### Principal Findings

This study demonstrates the acceptability of a machine learning–based predictive screener designed to support perinatal depression prevention. Predictive screening is an emerging field, and, to the best of our knowledge, this is the first study to examine receptivity to a predictive mental health screener, to query pregnant women about their desired preventive care options, and to examine differences in desired resources by predicted risk level. While machine learning algorithms are being newly applied to mental health prediction, their ultimate success depends on adoption into clinical workflow, which requires patient engagement and a feasible implementation process [29]. Here, we highlight an approach to evaluating receptivity to such predictive screeners while testing the feasibility of sending the screener through our health care system’s digital patient portal.

In this study, pregnant women who completed the screener largely reported positive screener experiences, with over 90% agreeing that it was clear, easy, and comfortable to respond to and that they had no concerns about responding honestly. More than 80% thought it would be helpful to know their risk of developing depression symptoms during pregnancy, regardless of their risk score. Those at risk of developing moderate to severe depression were slightly more likely to report feeling uncomfortable sharing their honest responses with an obstetric health care provider, suggesting that the screener questions touch on risk factors that are challenging to disclose. Perceived benefits and risks of the screener were similar between the no- or minimal-risk and at-risk groups, with 2 exceptions: those at risk of developing depression reported greater value in the screener for helping them understand contributors to their mood. They also reported greater fear about being judged by their provider. These anticipated challenges may be grounded in stigma and prior experiences of discrimination [30,31].

Deploying the screener through the patient portal reached a little more than one-third of the eligible pregnant women receiving prenatal care in the participating obstetric practices. This suggests that in-person screening may need to be offered as part of a clinical workflow to deploy the screener effectively to a majority of the patient population. However, among those who initiated the screening survey, engagement was encouraging, with almost 90% of participants completing the entire screening process. Thus, if the eligible patient reach is optimized through multiple outreach channels, the screener could have a high completion rate.

Survey participants identified specific perceived benefits and concerns associated with learning about being at risk of developing depression symptoms. The desire for therapy, whether in person or through an app, is reflective of the current best practices for preventive care, which include cognitive behavioral therapy and interpersonal therapy. Notably, participants also reported a desire for exercise opportunities and support groups, echoing research showing the protective effects of physical activity and social support on depression [18,21,32,33]. Child care was highly desired among the study population at risk of developing moderate to severe depression, suggesting that direct tangible support may impact the trajectory of depression risk. Financial stressors during pregnancy have previously been associated with poor outcomes for both mothers and infants. Findings from recent studies of guaranteed income programs for perinatal women show promising evidence of improvements in maternal mental health and infant developmental outcomes associated with alleviating financial stress [32,34-36].

This study contributes to a burgeoning literature on the importance of providing preventive mental health care during pregnancy and the postpartum period [37]. The perinatal period is uniquely vulnerable to depression due to a combination of hormonal changes, novel stressors (eg, pregnancy-related anxiety), and environmental factors [38]. However, up to half of the cases remain undiagnosed [39]. Although women without a psychiatric history are at lower baseline risk, they are more likely to be overlooked when they do experience depression, with lower rates of both depression screening and referral to care [40]. One solution to address the lack of diagnosis is more frequent, routine depression screening [13]. Care navigators dedicated to monitoring mental health symptoms throughout pregnancy, for example, have been shown to improve depressive symptoms among the patients they serve [41,42]. However, to date, there have been no screening tools available to support those professionals in identifying risk among individuals not actively reporting depressive symptoms [11]. If successfully implemented, predictive screeners could make the work of such care navigators more targeted and efficient.

### Limitations

This study has several limitations. Eligible participants were limited to those who could be contacted through an active patient portal. Although most of our prenatal patients have an active patient portal account, it is possible that these portals are not frequently accessed. Other studies suggest that individuals with limited health literacy are less likely to use patient portals to access health information [43-45]. Thus, the moderate response rate may introduce selection bias if individuals more familiar with portal-based screening were more likely to participate, whereas those with lower health literacy were less likely to engage. There were some differences in responses by participant risk level. Although these differences seem intuitive (eg, participants with greater depression risk were more likely to find the screener helpful in identifying influences on mood yet were more concerned about potential stigma from providers), they should be interpreted with caution given the modest sample size of the at-risk group and the number of comparisons conducted. The study sample was generally representative of the patient population of the academic medical system in which it was conducted, and the survey was developed using a patient-centered design approach with a diverse population. However, the results cannot be generalized to other health care systems in the United States or to clinics with more diverse patient populations. While the screener showed high accuracy within the sample on which it was built [17], further work is needed to establish independent external validation. Additional research must address how to best implement this screener within our health care system, including a testing workflow to fully close the loop on preventive care. A future goal would be to conduct validation and acceptability research of the screener in non-US populations.

### Conclusions

This study is a first step toward understanding how a machine learning–based prevention-focused predictive depression screener can be integrated into a clinical workflow for preventive care. We found that only one-third of the eligible pregnant women in our clinics completed the screener via their digital patient portal. Ultimately, deploying preventive screening tools will require a hybrid digital and in-person screening approach to comprehensively screen all pregnant women who may be at risk of developing perinatal depression. Our risk screener was designed to identify individuals who may need additional evaluation and care from a trained provider. As such, it is intended as a supplement to current prenatal care, allowing for earlier identification of those at risk rather than serving as a replacement for professional mental health evaluation. Our findings suggest that the screener was acceptable to those who completed it. Moreover, participants voiced clear preferences for certain forms of preventive care in response to learning their risk. Participants’ preference for receiving predictive depression screening results through the patient portal suggests a promising approach to expanding access to mental health assessment among individuals already engaged with digital health platforms. Our findings also suggest some next steps on how to inform the clinical workflow to support a more personalized approach to preventive care in pregnancy, leveraging the most endorsed preventive care resources (eg, counseling, peer support groups, exercise, and childcare support) to address the individual risk factors identified by the predictive screener.

## Abbreviations

STROBE: Strengthening the Reporting of Observational Studies in Epidemiology
